# Influence of Molecular Structures on Fluorescence of Flavonoids and Their Detection in Mammalian Cells

**DOI:** 10.3390/biomedicines10061265

**Published:** 2022-05-28

**Authors:** Ranjit De, Kyung Won Jo, Kyong-Tai Kim

**Affiliations:** Laboratory of Molecular Neurophysiology, Department of Life Sciences, Pohang University of Science and Technology (POSTECH), Pohang 37673, Korea; deranjit@postech.ac.kr (R.D.); kwjo0201@postech.ac.kr (K.W.J.)

**Keywords:** antioxidants, flavonoids, neuroblastoma cells, fluorescence, diphenylboric acid 2-aminoethyl ester, aluminum chloride

## Abstract

Flavonoids are being increasingly applied for the treatment of various diseases due to their anti-cancer, anti-oxidant, anti-inflammatory, and anti-viral properties. However, it is often challenging to detect their presence in cells and tissues through bioimaging, as most of them are not fluorescent or are too weak to visualize. Here, fluorescence possibilities of nine naturally occurring analogous flavonoids have been investigated through UV/visible spectroscopy, molecular structure examination, fluorescent images in mammalian cells and their statistical analysis employing aluminum chloride and diphenylboric acid 2-aminoethyl ester as fluorescence enhancers. It is found that, in order to form a stable fluorescent complex with an enhancer, flavonoids should have a keto group at C4 position and at least one -OH group at C3 or C5 position. Additionally, the presence of a double bond at C2–C3 can stabilize extended quinonoid structure at the cinnamoyl moiety, which thereby enhances the complex stability. A possible restriction to the free rotation of ring B around C1′–C2 single bond can contribute to the further enhancement of fluorescence. Thus, these findings can act as a guide for distinguishing flavonoids capable of exhibiting fluorescence from thousands of their analogues. Finally, using this technique, flavonoids are detected in neuroblastoma cells and their time course assay is conducted via fluorescence imaging. Their cellular uptake efficiency is found to be high and differential in nature and their distribution throughout the cytoplasm is clearly detected.

## 1. Introduction

**Figure 1:**
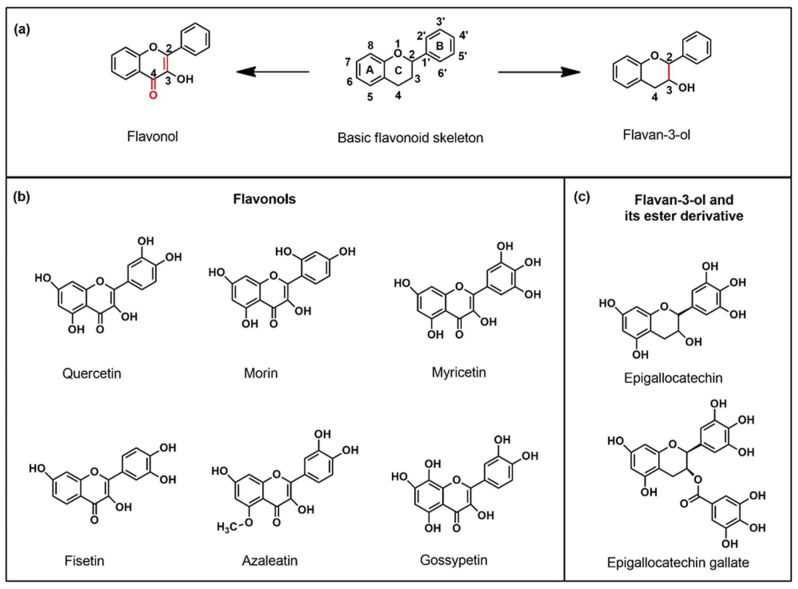
(**a**) Representative structure of basic flavonoid skeleton (phenylbenzopyran) along with its two subclasses used in this study—flavonol and flavanol demonstrating the differences at C4 position and the bond between C2 and C3. (**b**) Chemical structures of six flavonols (quercetin, morin, myricetin, fisetin, azaleatin, gossypetin) and (**c**) flavan-3-ol (epigallocatechin) and its ester derivative (epigallogatechin gallate) used in this study.

Flavonoids are naturally occurring polyhydroxy phenolic compounds found richly in tea, wine, flowers, fruits, roots, stem, bark, grains and vegetables, and they have significant potential in treating diseases such as cancer, diabetes, allergies, inflammation, neurodegeneration, viral infection, etc. [1,2,3]. They are also known as secondary metabolites of plants and cannot be synthesized by animals, or the microbiota present in their guts. The general chemical structure of flavonoids can be presented as a skeleton of a 15-carbon system with two phenyl rings and one heterocyclic ring with an oxygen atom embedded in it. This 15-carbon skeleton is often abbreviated as C6-C3-C6 [4]. Based on their molecular structures, these compounds can be classified into flavones, flavanones, flavanols, flavonols, isoflavones, chalcones and anthocyanidins [5]. Flavonoids have antiaging properties along with the ability to ameliorate learning and memory [6,7,8,9]. Recently, these compounds have attracted increased attention in treating several neurodegenerative diseases (NDs) [10,11,12,13,14]. They have been found to subdue microglial activation and modulate inflammatory process in the central nervous system (CNS) [15]. Furthermore, they can act as metal chelating agents, potential antioxidants, and free radical scavengers. These molecules have excellent therapeutic potential to prevent cancer via reactive oxygen species-mediated pathways [16]. Investigations have also shown that flavonoids can play a pivotal role in regulating oxidative stress in cells by fighting off free radicals [17]. Due to such free-radical scavenging properties, flavonoids are foreseen as promising for treating NDs via reactive oxygen species (ROS) control [18]. There are various food products rich in flavonoids, such as, red wine, tea, coffee, berries, apples, citrus fruits, soy products, cherries, grapes, onions, parsley, black beans, arugula, cowpeas, oregano, potato, corn flour, aubergine, plum, tomato, tofu, etc. [19,20]. Within a class, flavonoids differ in number and position of substitution in the aromatic rings A and B, while different classes of flavonoids differ in the level of oxidation and the pattern of substitution in the heterocyclic ring C, which remains fused in between the rings A and B (Figure 1a). The basic flavonol structure can also be presented as the combination of the benzoyl and cinnamoyl moieties, as shown in Figure S1.

NDs affect the structure and functions of specific neurons in central or peripheral nervous system, eventually leading to their death [21,22,23]. Birth and death of neuron cells are an intrinsic phenomenon during their development period; however, adult (postmitotic) neurons cannot reproduce and, hence, it is necessary for them to have long life to maintain accurate neural circuits [24,25]. Death of adult neurons caused by NDs can lead to the progressive degeneration of the brain. The most concerning NDs are Alzheimer’s, Parkinson’s, Amyotrophic Lateral Sclerosis (ALS), Huntington’s, etc., and the number of affected people suffering from these diseases is steeply rising. As the span of human life increases, which is currently ~3 years per generation, the challenges of halting the progress of NDs are soaring [26]. As is the case with various other risk factors, such as stroke, tumor and cancer in brain, and exposure to radiations, the excessive oxidative stress in neuronal cells is also detrimental and results in devastating loss of motor and cognitive functions of brain [27]. Oxygen is vital for life; however, disrupted balance between pro-oxidant/antioxidant homeostasis results in uncontrolled production of ROS such as super oxides (O_2_^•−^), hydroxyl radical (^•^OH), hydrogen peroxide (H_2_O_2_) and nitric oxide (NO), which induce high level of cellular stress [28,29]. Neuron cells are highly susceptible to degeneration caused by ROS. Horvathova et al. have shown that flavonoid molecules can protect deoxyribonucleic acid (DNA) against the action of ^•^OH radicals generated by H_2_O_2_ [30]. Similarly, these molecules can also nullify the oxidative stress caused by ROO^•^, NO^•^, etc. Being polyhydroxyphenols, they can donate protons from their hydroxyl groups to the radical species and thereby abolish their activities [12]. There are various flavonoid molecules which can also inhibit advanced glycation end-product (AGE) formation and thereby can prevent health disorders such as atherosclerosis, nephropathy, retinopathy, and neuropathy. Many flavonoid compounds are reported to show antiglycation activity [31,32,33]. Furthermore, it is known that matrix metalloproteinase (MMPs) can be activated by oxidative stress or AGE and can cause progressive damage to the extracellular matrix components, such as proteoglycans, collagen, etc. Since flavonoids are well-known anti-oxidants and can effectively reduce AGE, some of them are reported to inhibit MMP-1 and MMP-13 [34].

Recently, some of the flavonoids have been reported to permeate through blood–brain barrier (BBB), the semipermeable physiological membrane, and reach the neuronal cells. For example, Figueira et al. have demonstrated that these small molecules can have the potential to employ neuroprotective activities and thereby alleviate NDs, as they can cross the BBB to reach brain cells and modulate the microglia-mediated inflammation [35]. In another study, Youdim et al. showed that their permeation capability is consistent with their lipophilicity [36]. Thus, these compounds are becoming potential candidates to counteract the progression of neurodegeneration. Hence, flavonoids are in high demand to be used for further investigations against all these diseases and abnormal physiological state. However, it is highly challenging to detect them in the neuronal cells, as most of these compounds are not fluorescent, while some others are very weak to be visualized. Additionally, their concentration in the cellular milieu is often very low due to their restricted permeation through the BBB, and the interference of the cellular materials makes it further difficult to detect. Because of these challenges, the pharmacodynamics and pharmacokinetics of these compounds, specifically in mammalian cells, remained underexplored. Thus, a clear insight to correlate molecular structures of flavonoids to their fluorescence is essential. This will help to understand the reasons behind the non-fluorescent nature of many other flavonoids, which is at present a major obstacle to visualize their presence in mammalian cells and tissues. Such insights will also contribute to developing experimental techniques to enhance their fluorescence and detect via visualization.

To overcome this issue, various attempts are being made by researchers to explore relationships between the molecular structures of flavonoids and their fluorescence; however, a concrete understanding concerning establishing a universal principle has not yet been obtained as there are thousands (~8000) of naturally available flavonoids [37,38,39]. Moreover, the majority of the studies have dealt with plant cells and tissues, whereas their investigations in mammalian cells remained limited, especially studies involving neuronal cells are yet to flourish [40,41,42]. To identify the presence of flavonoids in cells and tissues, monitoring their fluorescence is the most popular method in practice. However, the fluorescence of flavonoids in physiologically relevant concentrations is extremely low or absent and, hence, they cannot be observed by a fluorescent microscope unless they are enhanced. Additionally, some of the flavonoids, despite their biomedical importance, could not be studied well as even after the use of fluorescence enhancers, the intensity is not sufficient to be detected or visualized. On the other hand, to enhance the fluorescence intensity, conjugation of large fluorescent moieties to the flavonoids increases their molecular size and incorporates the jeopardy of altering their inherent properties as well as permeation through physiological barriers; for example, their permeation through the BBB, skin, mucosal membrane, etc., can become restricted. For example, it is highly challenging for therapeutics with a molecular weight greater than 500 Da to pass through the BBB [43]. Even though, mass spectrometry, NMR, HPLC, etc., techniques can be applied to quantify flavonoids from the tissue extracts, visualization of these compounds is important to obtain a better understanding of the functions of these molecules at the cellular level. Thus, studies involving uptake of flavonoids in mammalian cells and tissues remained limited, particularly in neuronal cells.

In this work, two different fluorescence-enhancing molecules, aluminum chloride and diphenylboric acid 2-aminoethyl ester (DPBA), are employed in order to investigate their interactions with flavonoids. The basic molecular skeleton (phenylbenzopyran) of flavonoids, along with two of their classes (flavonol and flavan-3-ol) are used in this study [2,5]. The correlation between the molecular structure of flavonoids and their fluorescent property has been explored via UV-vis spectroscopy, molecular structure investigation, and fluorescence image analysis. The uptake of several flavonoids by neuroblastoma cells has been visualized through fluorescent images and their fluorescence enhancement inside the neuronal cells is presented. These molecules formed flavonoid-enhancer complexes and enhanced fluorescence, keeping the inherent molecular structure and function of flavonoids intact. Along with the molecular mechanism of fluorescence enhancement for some molecules, possible reasons for why the others failed to show fluorescence have also been discussed. The results have been compared with parallel experiments without the biological cells. Finally, this understanding is applied to investigate the time-course assay of different flavonoids that were taken up by the neuroblastoma cells.

## 2. Materials and Methods

### 2.1. Materials

Diphenylboric acid 2-aminoethyl ester (DPBA, 97%, Sigma-Aldrich, Burlington, VT, USA), aluminum chloride (99%, Sigma-Aldrich, Burlington, VT, USA), dimethyl sulfoxide (DMSO, 99.9%, Biopure Reagents, Minden, NV, USA), ethanol (99.5%, Sigma-Aldrich, Burlington, USA), quercetin (95%), morin hydrate (≥95–≤100%), myricetin (≥98%), epigallocatechin 3-gallate (≥95%), fisetin (≥90%), paraformaldehyde (95%), chlorotrimethylsilane (CTMS, ≥99%), acetonitrile (≥99.9%), imidazole (anhydrous, ≥99%), Hoechst for nucleus staining, and phosphate-buffered saline are purchased from Sigma-Aldrich, Burlington, USA. Gossypetin (98%) and azaleatin (97.5%) are procured from BOC Sciences, New York, NY, USA, while epigallocatechin (98.2%) is from Avachem, San Antonio, TX, USA. Human (SH-SY5Y) and mice (N2a) neuroblastoma cells are obtained from Korea Cell Line bank. Dulbecco’s Modified Eagle’s Medium (DMEM), fetal bovine serum (FBS), and penicillin streptomycin were obtained from Gibco, Waltham, MA, USA. UV-vis absorbance scan measurements were carried out using a Tecan infinite 200 Pro. The in vitro fluorescence images were obtained using a Zeiss Axioplan 2 upright fluorescence microscope while a Olimpus confocal microscope run by a software FV31S was used to obtain confocal images. The images were processed and analyzed using the software ImageJ (Bethesda, MD, USA). The Dako fluorescence mounting media was purchased from Agilent, Santa Clara, CA, USA.

### 2.2. Methods

#### 2.2.1. Sample Preparation for UV Absorption Spectroscopy and In Vitro Imaging

To prepare samples for UV absorption spectroscopy [44], 5 μL of flavonoid solution (100 mM) prepared in DMSO is added to 5 μL of 0.5% ethanoic solution of DPBA (or 0.1 M aqueous AlCl_3_) [3,45]. For in vitro imaging, a drop of (1 μL) of this solution is taken on silanized glass slide. The purpose of silanization of the glass slide is to prepare hydrophobic surface on it so that the spreading of the flavanol-DPBA or flavanol-AlCl_3_ drop can be minimized during imaging. The final concentration of flavonoids used is 100 μM. Silanization of microscope slides is carried out following the procedure established by Szkop et al. [46]. Briefly, slides are soaked in 5% detergent solution (150 mL), sonicated for 10 min, and cleaned with copious amounts of ultrapure water. These slides are then sonicated in methanol for about 10 min and again washed with ultrapure water followed by complete drying using an air gun before proceeding for silanization. The thoroughly cleaned slides are then dipped in a jar containing imidazole (0.54 M) and CTMS (3.3% *v*/*v*) in acetonitrile (150 mL), covered with a lid and heated for 3 h at 45 °C. These silanized slides are transferred to methanol and sonicated for 10 min followed by the transfer to ultrapure water and rocked for 5 min. The slides are finally dried and stored in a container sealed using parafilm for further use. For the imaging, a small drop (~1 μL) of flavonoid-DPBA (or flavonoid-AlCl_3_) was taken on this hydrophobic glass surface and their appearance was recorded using a digital camera. Fluorescent images of these drops were also recorded using a fluorescence microscope (Zeiss microscope Axioplan 2, Ex 470 nm–Em 525 nm).

#### 2.2.2. Cell Culture and Seeding

Neuroblastoma cells (N2a and SH-SY5Y) were grown in DMEM media supplemented with 10% FBS and 1% penicillin streptomycin in 100 mm cell culture dishes at 37 °C and 5% CO_2_ [23,47,48]. Cells are seeded on glass chips placed in a 12-well plate for uptake experiments and DPBA staining. About 1 × 10^5^ cells were seeded in each well containing 1 mL of the media and incubated for 24 h at 37 °C and 5% CO_2_ with humidified air.

#### 2.2.3. Flavonoid Treatment

Separate solutions of each flavonoid, namely, quercetin, morin hydrate, myricetin, epigallocatechin 3-gallate, fisetin, azaleatin, gossypetin, and epigallocatechin, are prepared in DMSO (100 mM) to treat the cells. Then, 1 μL of flavonoid solution (100 mM) is added to the media (1 mL) in one of the centrifuge tubes and vortexed well for mixing. This medium is then treated to each well that has cells on a glass chip after the complete removal of previous media from the well. To prepare a sample for control experiment, 1 μL of DMSO (without flavonoid) was mixed with media and treated to cells following the same procedure. Here, the final concentration of the flavonoids is 100 μM. These samples are then incubated for 1 h to allow the cells to uptake the flavonoids. Thus, a total of 9 samples were prepared and each sample is in three replicates. To carry out a time-course assay of different flavonoids in the neuroblastoma cells, the incubation period was varied (60 m, 30 m, 15 m, 5 m, 1 m, 30 s and 15 s).

#### 2.2.4. Cell Fixation, Staining and Imaging

##### Cell Fixation

The media of cells cultured in 12-well plate are discarded and the cells are washed three times by PBS (pH 7.4). Once washing is complete, each glass chip is transferred to another 12-well plate containing 1 mL of 4% paraformaldehyde prepared in PBS added to each well (i.e., 1 mL per well) and incubated for 40 min at room temperature [47]. The cells are then carefully washed by PBS three times.

##### Staining with DPBA, Aluminum Chloride

To stain the cells by DPBA or aluminum chloride, 0.5% DPBA in ethanol or aqueous aluminum chloride solution (0.1 M) was dropped gently on each chip [3,45]. These chips are then incubated at room temperature for 10 min. These cells were washed by PBS following the process used earlier. These steps were conducted in a dark room.

##### Nucleus Staining by Hoechst and Sample Preparation for Imaging

Hoechst solution (0.4 μg mL^−1^) was prepared in PBS [49]. Each chip is treated with Hoechst solution and kept in the dark for 10 min to stain the nucleus. These chips are then washed by PBS three times. Washed chips are then mounted on clean slide glass with the mounting media. These slides are stored in a dark place at 4 °C till the mounting media solidified. These samples are then used for imaging studies. Fluorescence microscopic images are acquired using Nikon Eclipse 90i, Ex 470 nm–Em 525 nm and 20× and 60× objective lenses.

#### 2.2.5. Statistical Analysis

All statistical analyses were performed using GraphPad Prism version 9.0. The significance of differences was assessed by one-way ANOVA followed by the Tukey’s multiple comparison tests. A *p*-value of *p* < 0.05 was considered to represent a significance. All data are presented as mean ± SEM.

## 3. Results

The two classes of flavonoids considered for the study differ according to the presence or absence of C2–C3 double bond and a double bonded oxygen at the C4 position of ring C, while the analogous polyphenol molecules differ according to the position and number of substitutions, namely –OH, and –OCH_3_ groups in rings A and B (Figure 1b). Epigallocatechin gallate, the ester derivative of epigallocatechin and gallic acid, is chosen to investigate the influence of substitution at position 3 on the fluorescence of this molecule when the C2–C3 double bond is absent (Figure 1c). The results of fluorescence enhancement both outside the cells as well as in human and mice neuroblastoma cells induced by staining with AlCl_3_ and DPBA are discussed below, along with the time dependent cellular uptake.

### 3.1. Absorption Spectroscopy

**Figure 2:**
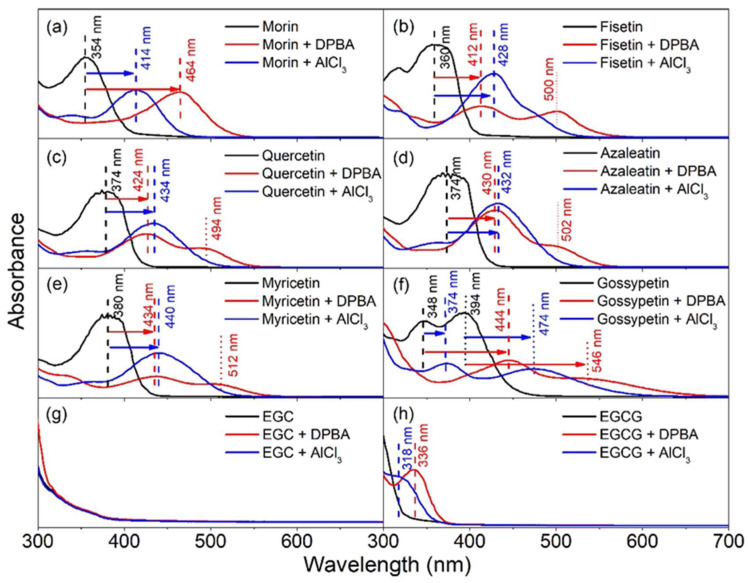
UV-visible spectra of flavonoids showing absorption bands and their shifts after they were treated with DPBA and AlCl_3_. Impact of DPBA and AlCl_3_ into the band shift of (**a**) morin, (**b**) fisetin, (**c**) quercetin, (**d**) azaleatin, (**e**) myricetin, (**f**) gossypetin, (**g**) epigallocatechin (EGC) and (**h**) epigallocatechin gallate (EGCG). The absorption spectra of DPBA and AlCl_3_ in DMSO in absence of flavonoids are provided in the Supplementary Materials in Figure S2. Dashed and dotted lines represent the positions of absorption peaks before and after their shifts, respectively.

**Figure 3:**
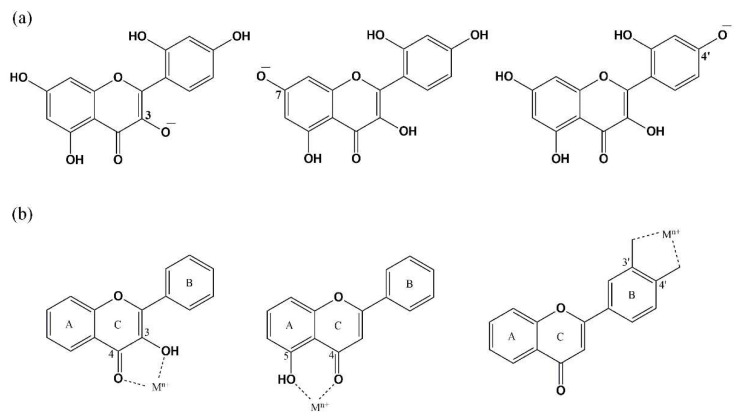
(**a**) The readily ionizable sites (C3, C7, and C4′) of flavonoids. (**b**) Presence of -OH or =O groups at the nearby C-atom favors the complexation process.

**Figure 4:**
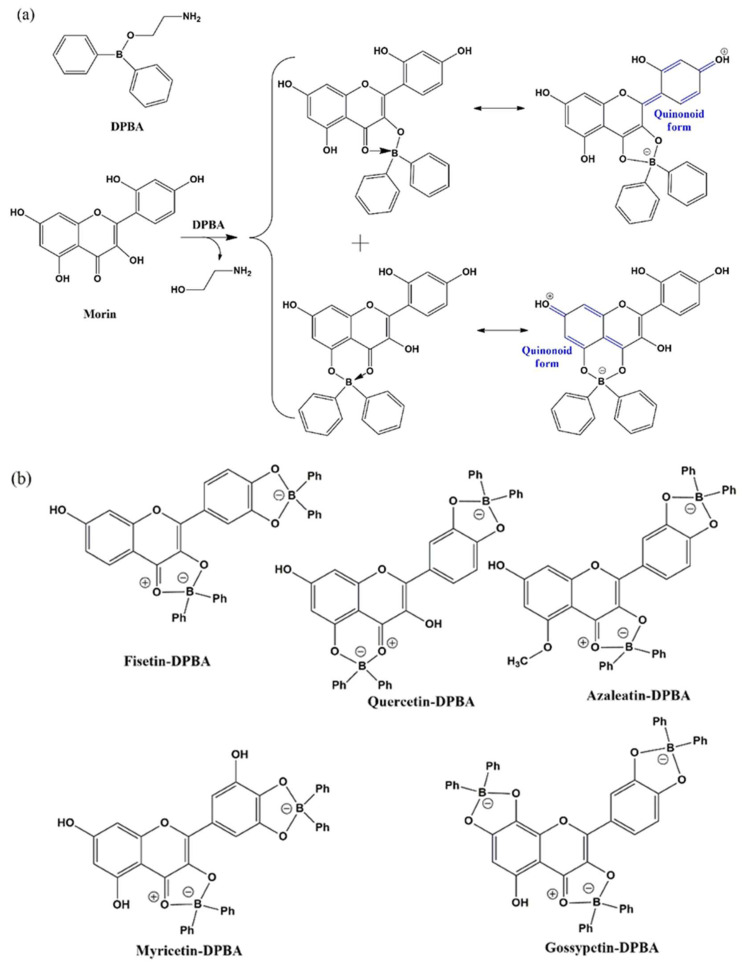
(**a**) A schematic presentation of the complexation of DPBA with morin and the plausible quinonoid forms. (**b**) The plausible molecular structures of complexes formed on treatment of fisetin, quercetin, azaleatin, myricetin and gossypetin with DPBA.

Flavonoids exhibit UV absorption properties and, hence, the impact of the addition of the complexing agent (DPBA or AlCl_3_) into the flavonoid solutions outside biological cells is initially investigated by analyzing their absorption spectra (Figure 2). The absorption spectra of DPBA and AlCl_3_ in DMSO in absence of flavonoids showed no peak and are considered as references. These spectra are provided in the Supplementary Materials (Figure S2). Apart from epigallocatechin, notable bathochromic shifts in absorption spectra have been observed for all other flavonoids investigated here. The UV absorption profile of morin (in absence of enhancers) showed a maximum at 354 nm, which can be attributed to the absorption of photons by the cinnamoyl moiety of the molecule that involves rings B and C (Figure 1a) [50,51,52]. However, when this flavonol is treated with AlCl_3_ or DPBA, both the cases have exhibited bathochromic shifts of the original absorption band (354 nm) by 60 nm for AlCl_3_ and 110 nm for DPBA (Figure 2a, Table 1). The bathochromic shift values of all the absorption peaks caused by the AlCl_3_ or DPBA treatments of flavonoids investigated in this study are summarized in Table 1. The band position, intensity and shape of absorption spectra can be influenced by dipole–dipole interaction, H-bonding, and ionic interactions with the surrounding molecules. Hence, it is evident that the shifts are caused by the interactions between flavonoids and the added enhancer AlCl_3_ (or DPBA) and the interaction is predominantly at the C ring involving the C=O group in the case of flavonols [10]. However, interaction with the other rings cannot be negated as the bands corresponding to such interactions are also present, which is very similar to the observation reported by Matteini et al. for a different flavonoid molecule rutin [44,53]. In this study, bathochromic shifts of the absorption band of morin (for example) from 354 nm to 414 nm caused by the AlCl_3_ treatment and to 464 nm by DPBA treatment suggest the formation of complexes or chelates. The protons of hydroxyl groups of the flavonoid molecules at C3, C7, and C4ʹ positions are readily ionizable as presented in Figure 3a [54]. Furthermore, the -OH or C=O moieties located close to these C3, C7, and C4ʹ positions can favor the interaction between flavonoid molecules and enhancers as demonstrated in Figure 3b. It is also noteworthy that the presence of C2–C3 double bond can facilitate the delocalization of π-electrons all the way to the C4 position from ring B, resulting in an increase in the electron cloud around the oxygen atom of the C=O group. This can favor the electronic transition between π-type molecular orbitals (π → π* transition) and enhance the polarity of the molecule favoring the complexation. Additionally, the delocalization of π-electrons involving ring B and C can favor the formation of stable quinonoid structures, as demonstrated in Figure 4a. A similar quinonoid structure can also be formed involving rings A and C. Considering morin as an example, both quinonoid structures are presented in Figure 4a. A schematic presentation of similar complexes formed with AlCl_3_ is presented in Figure S3 of the Supplementary Materials. These structural properties can facilitate the formation of stable complexes. Plausible molecular structures of complexes formed between DPBA and other flavonols are presented in Figure 4b. Earlier, Biler et al. proposed that the absorption spectra of flavonoids can be influenced by various structural motifs, such as deprotonation states and population of -OH groups at the B ring, and the substituent at the C3 position [55]. Additionally, the presence of any other factors in the flavonoid molecules, such as electron withdrawing or donating groups can also contribute to influencing the extent of complexation.

In this study, epigallocatechin did not show any absorbance peak in the investigated region under the adopted experimental conditions and, hence, no bathochromic shift could be noted. It is known that the presence of -OH groups in ring B can contribute to the absorbance peak; however, due to the absence of C2–C3 double bond delocalization of the π-electrons were not favored, neither the formation of any quinonoid structure could take place. Thus, even if the complexation occurs involving the ortho-hydroxyl groups of ring B, it would be unstable or labile resulting in a failure to introduce fluorescence to this molecule. Furthermore, there is no keto group at the C4 position; as a result, no stable complexation can take place there, even when the -OH group is present at the C5 position. Therefore, these phenomena can be attributed to the absence of the absorption peak at the investigated wavelength region. This shows that the presence of -OH groups along with the C2–C3 double bond and the keto group at the C4 position plays crucial role in the complex formation. Based on this insight it is also realized why epigallocatechin gallate did not show any pronounceable bathochromic shift which is due to the absence of C2–C3 double bond. However, this molecule has a gallate moiety linked to the oxygen present at the C3 position and this moiety has a double bonded oxygen atom outside the benzene ring, which can take part in the formation of a quinonoid structure but which could not facilitate the complexation process adequately as there is no available -OH group at the vicinity. Nevertheless, this molecule has six ortho -OH groups which may form labile complexes, and this could be attributed to the absorption peaks that appeared below 350 nm.

### 3.2. In Vitro Study

**Figure 5:**
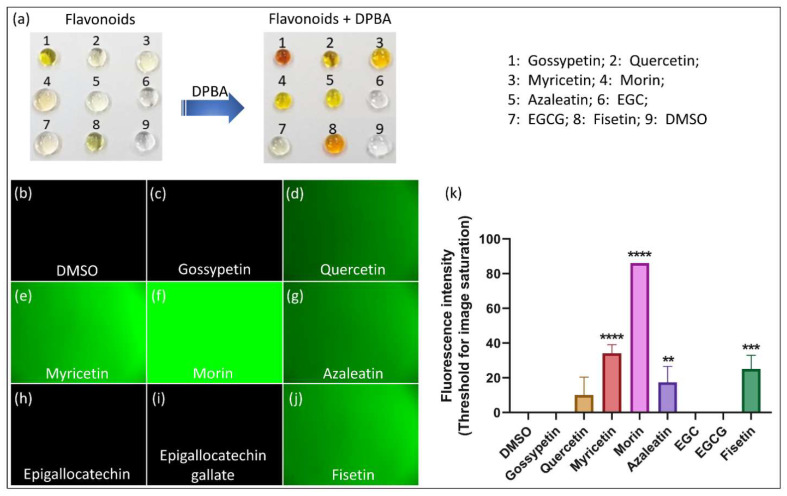
In vitro investigation of fluorescence response of flavonoids after interaction with DPBA. (**a**) Images of the flavonoid droplets on silanized glass slide before and after their treatment with DPBA. The concentration of flavonoids prepared in DMSO is 100 mM while the concentration of DPBA solution used for the treatment is 0.5% in EtOH. The 1:1 (*v*/*v*) mixture was dropped (~1 μL) on the silanized glass slide. (**b**–**j**) Optical microscopic images (Ex. 470 nm) of the flavonoids treated with DPBA where morin, myricetin, fisetin, azaleatin and quercetin exhibited fluorescence and (**k**) a comparison of their relative intensity. The error bars represent the mean ± SEM. **** *p* < 0.0001, *** *p* < 0.001, ** *p* < 0.01, one-way ANOVA followed by Tukey’s multiple comparisons test (**k**).

Fluorescence exhibited by the flavonoids investigated through the in vitro (without the biological cells) study is presented in Figure 5. As observed through the earlier presented UV absorption study, the flavonoid molecules morin, fisetin, quercetin, azaleatin and myricetin have shown their fluorescence activity when treated with DPBA. Similar findings are reproduced by these molecules in the case of AlCl_3_ treatment and presented in the Supplementary Materials (Figure S4). Thus, the above-mentioned findings regarding the structural requirement of flavonoid molecules to exhibit fluorescence have been confirmed through these in vitro investigations. This confirms that the presence of a keto group at the C4 position of ring C, hydroxyl group at the C3 or C5 position or both, along with the presence of a double bond at the C2–C3 position of flavonoids, can contribute to producing strong fluorescence when an enhancer is used.

**Figure 6:**
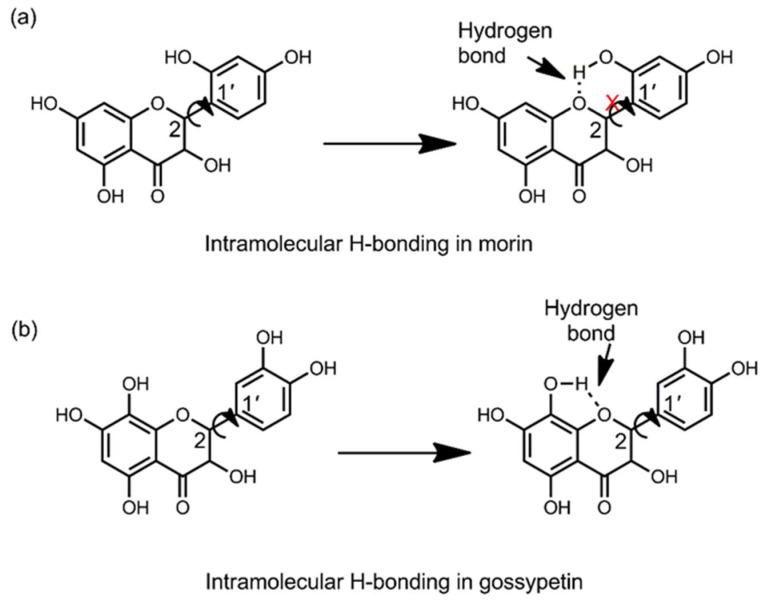
Possibility of intramolecular H-bonding in morin can restrict the free rotation of the cinnamoyl and benzoyl moieties around C1′-C2 single bond (**a**) while such contribution in gossypetin is not feasible (**b**).

Furthermore, it is noticed that among the flavonoids studied here, morin demonstrated the highest fluorescence intensity upon interacting with DPBA. A similar observation was noted by Hollman et al. where this molecule was treated with AlCl_3_ after conducting the high performance liquid chromatography to detect its fluorescence through post column chelation [56]. Such enhanced fluorescence intensity of morin can be attributed to the presence of its hydroxyl group at C2′, which can form an intramolecular H-bond between the H atom of this OH-group and the hetero atom oxygen at position C1 of ring C (Figure 6a). Once the H-bond is formed, it can restrict the free rotation of ring B around the C2-C1ʹ single bond and introduce rigidity (planar conformation) to the molecule, which thereby can enhance the stability of its quinonoid form, resulting in the enhanced fluorescence efficacy [53,57]. Thus, it can be noted that, in addition to the above-mentioned criteria of flavonoids that favor the formation of stable complexes with AlCl_3_ or DPBA, the presence of a hydroxyl group at the C2′ position of ring B can contribute to fluorescence enhancement facilitated by intramolecular interactions.

It was known that of the flavonoids, only the flavonols that contain a free hydroxyl group at position C3 or C5 and a keto group at position C4 can form complexes with AlCl_3_ giving rise to fluorescence [56]. However, this study has shown that in addition to these criteria, presence of the C2–C3 double bond and a hydroxyl group at the C2′ position of ring B can further facilitate fluorescence enhancement. Similarly, it is expected that presence of a hydroxyl group at the C8 position of ring A might favor the formation of an intra molecular H-bond between this -OH group and the hetero atom oxygen at the position 1 of ring C; however, it does not introduce any additional contribution to stabilize the molecule as the rings A and C are already fused with each other (Figure 6b). Instead, once the OH-group at C8 position is engaged in intramolecular H-bond formation, there is no ortho-hydroxyl group available for the OH-group at C7 position; hence, no stable complexation with fluorescent agent can take place at the A ring of gossypetin involving this hydroxyl group. Thus, it can be understood why gossypetin, despite having an extra hydroxyl group (C8 position) in comparison to that of quercetin, morin, fisetin and azaleatin, could not contribute by exhibiting sufficient fluorescence. It is also noteworthy that the presence of ortho dihydroxy groups in ring B can render higher degree of stability to flavonoid phenoxyl ions via the π-electron delocalization [58]. Hence, the presence of hydroxyl group at the C8 position could not contribute to fluorescent enhancement in gossypetin.

### 3.3. Detection of Different Flavonoids in Neuroblastoma Cells

**Figure 7:**
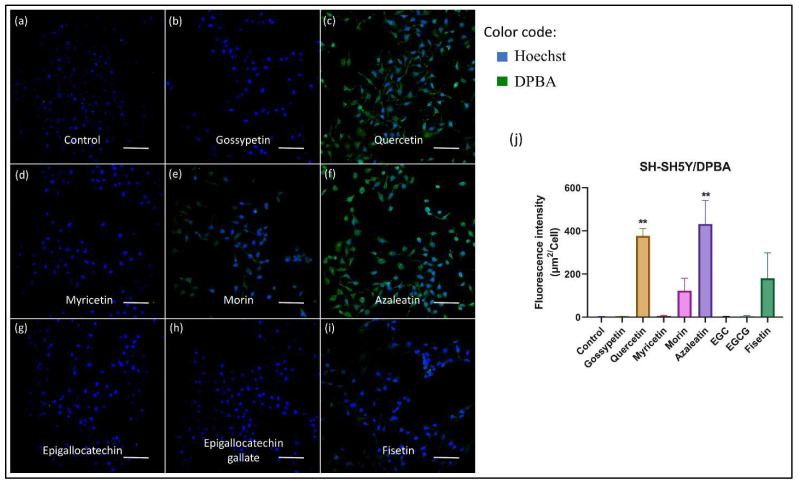
Fluorescence microscopic images (Ex. 470 nm) of human neuroblastoma (SH-SY5Y) cells after flavonoid uptake followed by DPBA treatment. Cells are treated with (**a**) DMSO (flavonoid absent, control), (**b**) azaleatin, (**c**) quercetin, (**d**) fisetin, (**e**) morin, (**f**) myricetin, (**g**) gossypetin, (**h**) epigallocatechin, and (**i**) epigallocatechin gallate. Flavonoid treatment (100 μM) is carried out for 1 h, while the enhancer DPBA solution (0.5% wt./*v*, in ethanol) is employed for 10 min. The scale bars represent 100 μm. Nucleus was stained using Hoechst. Among these, the azaleatin, quercetin, fisetin and morin treated cells showed fluorescence while it was not detectable in gossypetin, epigallocatechin, and epigallocatechin gallate treated cell. Panel (**j**) represents a comparison of fluorescence intensity exhibited by the flavonoid treated cells. The error bars represent the mean ± SEM. ** *p* < 0.01, one-way ANOVA followed by Tukey’s multiple comparisons test (**j**).

To investigate whether the fluorescence enhancement of flavonoids by DPBA (or AlCl_3_) complexation can be employed in mammalian cells, neuroblastoma cells (human and mouse) were imaged before and after DPBA (or AlCl_3_) treatment. Images of fluorescent detection of flavonoids taken up by human neuroblastoma (SH-SY5Y) cells are presented in Figure 7. The cells that are not treated with DPBA (or AlCl_3_) did not show any fluorescence (images not shown). Images of the SH-SY5Y cells parallelly checked by treating with AlCl_3_ are presented in Figure S5 of the Supplementary Materials. Here, we found that out of all the flavonoids tested, only morin, quercetin, and azaleatin showed fluorescence. Since no fluorescence was detected in the cells that were treated with flavonoids but not with the fluorescent agent DPBA (or AlCl_3_), it is obvious that these fluorescence enhancers can be used to detect flavonoids in neuroblastoma cells. Here, it is noteworthy that for myricetin, though the absorption peak shift is clearly visible (Figure 2e), the intensity was not that high, which is reflected in both the experiments conducted inside the cells, i.e., the fluorescence of myricetin could not be detected after its treatment with AlCl_3_ or DPBA (Figure 7d and Figure S5f). On the other hand, the fluorescence of fisetin is detected using the DPBA, while it did not exhibit fluorescence when treated with AlCl_3_ (Figure 7i and Figure S5e). Though detailed investigation is required to understand this behavior, it is apparent that the molecular structure of enhancers can play a significant role in the complexation process and fluorescence enhancement. In comparison to other flavonols, fisetin has a relatively lower number of -OH groups substituted to the ring A, which could be the reason that the extent of effective complexation with AlCl_3_ was less. Again, in gossypetin, there is an -OH group present at position C5 in ring A, which should favor the complexation; however, the presence of another -OH group at the position C8 can interfere with the stability of the gossypetin-enhancer complex, which might have caused it to be non-fluorescent. However, the cells treated with gossypetin, epigallocatechin, and epigallocatechin gallate, despite the use of the enhancers, did not show fluorescence, which is in line with the observation noted earlier through UV visible spectroscopic and in vitro (outside cells) studies. It is noteworthy that the order of fluorescence intensity observed in the earlier in vitro (outside cells) studies did not remain the same. This is because cells can have different uptake efficiency for different flavonoid compounds and, at the same time, the cellular materials can also differently interact with different flavonoids depending on the molecular structures of the flavonoids. Fluorescence microscopic images of mouse neuroblastoma cells (N2a) treated with DPBA as well as AlCl_3_, which are presented in the Supplementary Materials (Figures S6 and S7), have exhibited similar phenomena.

### 3.4. Time-Course Analysis

**Figure 8:**
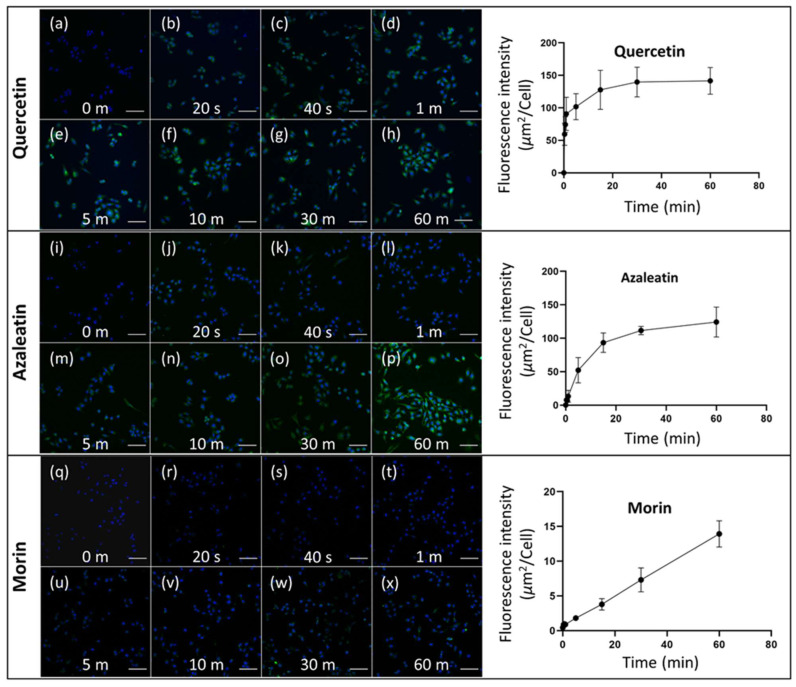
Fluorescent microscopic images of flavonoid uptaken SH-SY5Y cells treated with DPBA for various durations, (**a**–**h**) quercetin, (**i**–**p**) azaleatin and (**q**–**x**) morin. Increase in fluorescence intensity as a function of treatment time are presented for each flavo.

To carry out time-course analysis to investigate the cellular uptake, three representative flavonoids, namely quercetin, azaleatin and morin are considered. The presence of quercetin in the cytoplasm of SH-SY5Y cells is clearly visible after 1 min of incubation through sufficiently bright fluorescence image (Figure 8a–h), whereas to achieve similar level of accumulation of morin, it required about 10 min of incubation (Figure 8i–p). This shows that the uptake of these flavonoids by the neuroblastoma cells is fast enough. For morin, it is also observed that the molecules are sufficiently taken up by the neuroblastoma cells (Figure 8q–x); however, it did not show similar brightness even after the incubation duration of 1 h, as shown by quercetin and azaleatin. Nevertheless, it is clearly observed that with the increase in the duration of incubation, the intensity of fluorescence increases. These results imply that the differential accumulations of all the flavonoids occurred with the increase in duration of incubation. It is noteworthy that differences in flavonoid metabolism could also be one of the reasons to exhibit different fluorescence behavior in cells. This shows that the fluorescence enhancer, such as DPBA, can be used to monitor the uptake procedure of flavonoids by the animal cells and is sufficiently sensitive, as is the case for plant cells. However, it is observed that the fluorescence intensity of morin observed during the studies carried out outside the cells was the highest, whereas it is the lowest in the cells. Similar results have also been observed through Figure 7. This indicates that the uptake efficiency of morin into the cell is weaker than in other flavonoid compounds. It could also be possible that cellular materials are interacting with the flavonoids, and in this case, they may interact more efficiently with morin due to its specific molecular structure. This can interfere with the interactions between flavonoids and DPBA. To show the location of flavonoids inside cell more clearly, a magnified images of quercetin-treated single cell taken using the confocal microscope has been presented in Figure S8 [59]. This confirms the distribution of flavonoids inside neuroblastoma cells and are not confined to any specific region. Therefore, it is understood that this fluorescent probe can be used to visualize the presence of flavonoids in the cytosol of neuronal cells and the process is sensitive enough to carry out pharmacodynamic and pharmacokinetic studies.

## 4. Conclusions

In this work, the imaging possibilities of nine naturally occurring flavonoids are investigated using DPBA and AlCl_3_ as fluorescence enhancers. These enhancers have introduced fluorescence to some of the flavonoid molecules, whereas to others they could not. Possible reasons behind such phenomena have been explored in the light of molecular structures of the flavonoids. It is found that the presence of a keto group at the C4 position of ring C along with the presence of at least one -OH group at the C3 or C5 position are required to form the flavonoid-enhancer complexes to exhibit fluorescence. Here, it is understood that the presence of a double bond at the C2–C3 position of ring C can contribute to form extended quinonoid structure at the cinnamoyl moiety of flavonoids which thereby can contribute to form stable fluorescent complex. Restriction of free rotation of the B ring around C1′-C2 single bond can contribute to the enhancement of fluorescence intensity. It is also noted that the presence of a hydroxyl group at the C8 position could not contribute to the fluorescence enhancement. The fluorescence intensities of flavonoids inside the cell were found to be different to that outside the cell. It is understood that, initially, this could be due to different cellular uptake efficiencies for different flavonoids. Furthermore, biomolecules present in the cellular milieu can exhibit different extent of affinity to interact with the flavonoids with different molecular motifs. A detailed understanding on this requires further investigation. However, this phenomenon must be considered during the data analysis of where imaging of flavonoids in biological cells are involved. Thus, a systematic investigation on the molecular structural requirements of flavonoids that contribute towards efficient complexation with fluorescence enhancers are presented. The conclusions drawn from this study can guide researchers to predict the flavonoids that would exhibit fluorescence even prior to an actual experiment is conducted, which would otherwise be challenging to screen the huge number of naturally available flavonoids.

## Figures and Tables

**Table 1:** Bathochromic shifts of absorption peaks of the flavonoids induced by AlCl_3_ and DPBA.

Flavonoids	Peak Position (nm)	Peak Positions (nm) after Bathochromic Shift Induced by	
		AlCl_3_	DPBA
Morin	354	414	464
Fisetin	360	428	412, 500
Quercetin	374	434	424, 494
Azaleatin	374	432	430, 502
Myricetin	380	440	434, 512
Gossypetin	348	374, 474	444, 546
Epigallocatechin	-	-	-
Epigallocatechin gallate	-	318	336

## Supplementary Materials

The following supporting information can be downloaded at: www.mdpi.com/article/10.3390/biomedicines10061265/s1, Figure S1: A schematic presentation of the basic flavonol structure comprised of the benzoyl and cinnamoyl moieties; Figure S2: UV-visible spectra of DPBA and AlCl_3_ in DMSO showing no peaks in the absence of flavonoids; Figure S3: A schematic presentation of the complexation of AlCl_3_ with morin and the plausible quinonoid forms; Figure S4: In vitro Investigation of fluorescence intensity of the flavonoids after chelation with AlCl_3_ (a–i) and a comparison of their relative intensity (j); Figure S5: Fluorescence microscopic images of human neuroblastoma (SH-SY5Y) cells after flavonoid uptake followed by AlCl_3_ treatment. Cells are treated with (a) DMSO (flavonoid absent, control), (b) azaleatin, (c) quercetin, (d) fisetin, (e) morin, (f) myricetin, (g) gossypetin, (h) epigallocatechin, and (i) epigallocatechin gallate. Flavonoid treatment (100 μM) is carried out for 1 h, while the enhancer AlCl_3_ solution (0.01 M in water) is employed for 10 min. The scale bars represent 100 μm. Nucleus was stained using Hoechst. Among these, the azaleatin, quercetin, fisetin and morin treated cells showed fluorescence while it was not detectable in gossypetin, epigallocatechin, and epigallocatechin gallate treated cell. Panel (j) represents a comparison of fluorescence intensity exhibited by the flavonoid treated cells; Figure S6: Fluorescence microscopic images of mouse neuroblastoma (N2a) cells after flavonoid uptake followed by DPBA treatment. Cells are treated with (a) DMSO (flavonoid absent, control), (b) azaleatin, (c) quercetin, (d) fisetin, (e) morin, (f) myricetin, (g) gossypetin, (h) epigallocatechin, and (i) epigallocatechin gallate. Flavonoid treatment (100 μM) is carried out for 1 h, while the enhancer DPBA solution (0.5% wt./v, in ethanol) is employed for 10 min. The scale bars represent 100 μm. Nucleus was stained using Hoechst. Among these, the azaleatin, quercetin, fisetin and morin treated cells showed fluorescence while it was not detectable in gossypetin, epigallocatechin, and epigallocatechin gallate treated cell. Panel (j) represents a comparison of fluorescence intensity exhibited by the flavonoid treated cells; Figure S7: Fluorescence microscopic images of human neuroblastoma (N2a) cells after flavonoid uptake followed by AlCl3 treatment. Cells are treated with (a) DMSO (flavonoid absent, control), (b) azaleatin, (c) quercetin, (d) fisetin, (e) morin, (f) myricetin, (g) gossypetin, (h) epigallocatechin, and (i) epigallocatechin gallate. Flavonoid treatment (100 μM) is carried out for 1 h, while the enhancer AlCl_3_ solution (0.01 M in water) is employed for 10 min. The scale bars represent 100 μm. Nucleus was stained using Hoechst. Among these, the azaleatin, quercetin, fisetin and morin treated cells showed fluorescence while it was not detectable in gossypetin, epigallocatechin, and epigallocatechin gallate treated cell. Panel (j) represents a comparison of fluorescence intensity exhibited by the flavonoid treated cells. Figure S8: A magnified image of quercetin treated SH-SY5Y single cell stained with DPBA showing the distribution of quercetin inside the cell. The scale bar represents 10 μm.

## References

[1] Panche A.N., Diwan A.D., Chandra S.R. (2016). Flavonoids: An Overview. J. Nutr. Sci..

[2] Maher P. (2019). The Potential of Flavonoids for the Treatment of Neurodegenerative Diseases. Int. J. Mol. Sci..

[3] Ferrara B.T., Thompson E.P. (2019). A Method for Visualizing Fluorescence of Flavonoid Therapeutics in Vivo in the Model Eukaryote Dictyostelium Discoideum. Biotechniques.

[4] De Souza Farias S.A., Da Costa K.S., Martins J.B.L. (2021). Analysis of Conformational, Structural, Magnetic, and Electronic Properties Related to Antioxidant Activity: Revisiting Flavan, Anthocyanidin, Flavanone, Flavonol, Isoflavone, Flavone, and Flavan-3-Ol. ACS Omega.

[5] Kicinska A., Jarmuszkiewicz W. (2020). Flavonoids and Mitochondria: Activation of Cytoprotective Pathways?. Molecules.

[6] Tungmunnithum D., Tanaka N., Uehara A., Iwashina T. (2020). Flavonoids Profile, Taxonomic Data, History of Cosmetic Uses, Anti-Oxidant and Anti-Aging Potential of *Alpinia Galanga* (L.) Willd. Cosmetics.

[7] Structures C. (2015). Encyclopedia of Inflammatory Diseases. Encycl. Inflamm. Dis..

[8] Rendeiro C., Guerreiro J.D.T., Williams C.M., Spencer J.P.E. (2012). Flavonoids as Modulators of Memory and Learning: Molecular Interactions Resulting in Behavioural Effects. Proc. Nutr. Soc..

[9] Vauzour D. (2014). Effect of Flavonoids on Learning, Memory and Neurocognitive Performance: Relevance and Potential Implications for Alzheimer’s Disease Pathophysiology. J. Sci. Food Agric..

[10] Kasprzak M.M., Erxleben A., Ochocki J. (2015). Properties and Applications of Flavonoid Metal Complexes. RSC Adv..

[11] Brunetti C., Di Ferdinando M., Fini A., Pollastri S., Tattini M. (2013). Flavonoids as Antioxidants and Developmental Regulators: Relative Significance in Plants and Humans. Int. J. Mol. Sci..

[12] Treml J., Šmejkal K. (2016). Flavonoids as Potent Scavengers of Hydroxyl Radicals. Compr. Rev. Food Sci. Food Saf..

[13] Tyagi N., Song Y.H., De R. (2019). Recent Progress on Biocompatible Nanocarrier-Based Genistein Delivery Systems in Cancer Therapy. J. Drug Target..

[14] Tyagi N., De R., Begun J., Popat A. (2017). Cancer Therapeutics with Epigallocatechin-3-Gallate Encapsulated in Biopolymeric Nanoparticles. Int. J. Pharm..

[15] Spencer J.P.E., Vafeiadou K., Williams R.J., Vauzour D. (2012). Neuroinflammation: Modulation by Flavonoids and Mechanisms of Action. Mol. Asp. Med..

[16] Slika H., Mansour H., Wehbe N., Nasser S.A., Iratni R., Nasrallah G., Shaito A., Ghaddar T., Kobeissy F., Eid A.H. (2022). Therapeutic Potential of Flavonoids in Cancer: ROS-Mediated Mechanisms. Biomed. Pharmacother..

[17] Kang Y., Lee J.H., Seo Y.H., Jang J.H., Jeong C.H., Lee S., Jeong G.S., Park B. (2019). Epicatechin Prevents Methamphetamine-Induced Neuronal Cell Death via Inhibition of Er Stress. Biomol. Ther..

[18] Devi S., Kumar V., Singh S.K., Dubey A.K., Kim J.J. (2021). Flavonoids: Potential Candidates for the Treatment of Neurodegenerative Disorders. Biomedicines.

[19] Manach C., Scalbert A., Morand C., Rémésy C., Jiménez L. (2004). Polyphenols: Food Sources and Bioavailability. Am. J. Clin. Nutr..

[20] Kozłowska A., Szostak-Węgierek D., Mérillon J.M., Ramawat K. (2018). Flavonoids—Food Sources, Health Benefits, and Mechanisms Involved. Bioactive Molecules in Food.

[21] Chi H., Chang H.Y., Sang T.K. (2018). Neuronal Cell Death Mechanisms in Major Neurodegenerative Diseases. Int. J. Mol. Sci..

[22] Gorman A.M. (2008). Neuronal Cell Death in Neurodegenerative Diseases: Recurring Themes around Protein Handling: Apoptosis Review Series. J. Cell. Mol. Med..

[23] Song Y.H., De R., Lee K.T. (2021). Uptake of Polyelectrolyte Functionalized Upconversion Nanoparticles by Tau-Aggregated Neuron Cells. Pharmaceutics.

[24] Fricker M., Tolkovsky A.M., Borutaite V., Coleman M., Brown G.C. (2018). Neuronal Cell Death. Physiol. Rev..

[25] Stiles J., Jernigan T.L. (2010). The Basics of Brain Development. Neuropsychol. Rev..

[26] Poewe W., Seppi K., Tanner C.M., Halliday G.M., Brundin P., Volkmann J., Schrag A.E., Lang A.E. (2017). Parkinson Disease. Nat. Rev. Dis. Prim..

[27] Saxena S., Caroni P. (2011). Selective Neuronal Vulnerability in Neurodegenerative Diseases: From Stressor Thresholds to Degeneration. Neuron.

[28] Barnham K.J., Masters C.L., Bush A.I. (2004). Neurodegenerative Diseases and Oxidatives Stress. Nat. Rev. Drug Discov..

[29] Article R., Hwang O. (2013). Role of Oxidative Stress in Parkinson’s Disease. Exp. Neurobiol..

[30] Horváthová K., Novotný L., Vachálková A. (2003). The Free Radical Scavenging Activity of Four Flavonoids Determined by the Comet Assay. Neoplasma.

[31] Sampath C., Rashid M.R., Sang S., Ahmedna M. (2017). Green Tea Epigallocatechin 3-Gallate Alleviates Hyperglycemia and Reduces Advanced Glycation End Products via Nrf2 Pathway in Mice with High Fat Diet-Induced Obesity. Biomed. Pharmacother..

[32] Spagnuolo L., Posta S.D., Fanali C., Dugo L., De Gara L. (2021). Antioxidant and Antiglycation Effects of Polyphenol Compounds Extracted from Hazelnut Skin on Advanced Glycation End-products (Ages) Formation. Antioxidants.

[33] Zhou Q., Cheng K.W., Xiao J., Wang M. (2020). The Multifunctional Roles of Flavonoids against the Formation of Advanced Glycation End Products (AGEs) and AGEs-Induced Harmful Effects. Trends Food Sci. Technol..

[34] Ronsisvalle S., Panarello F., Longhitano G., Siciliano E.A., Montenegro L., Panico A. (2020). Natural Flavones and Flavonols: Relationships among Antioxidant Activity, Glycation, and Metalloproteinase Inhibition. Cosmetics.

[35] Figueira I., Garcia G., Pimpão R.C., Terrasso A.P., Costa I., Almeida A.F., Tavares L., Pais T.F., Pinto P., Ventura M.R. (2017). Polyphenols Journey through Blood-Brain Barrier towards Neuronal Protection. Sci. Rep..

[36] Youdim K.A., Dobbie M.S., Kuhnle G., Proteggente A.R., Abbott N.J., Rice-Evans C. (2003). Interaction between Flavonoids and the Blood-Brain Barrier: In Vitro Studies. J. Neurochem..

[37] Baldim J.L., De Alcantara B.G.V., Domingos O.D.S., Soares M.G., Caldas I.S., Novaes R.D., Oliveira T.B., Lago J.H.G., Chagas-Paula D.A. (2017). The Correlation between Chemical Structures and Antioxidant, Prooxidant, and Antitrypanosomatid Properties of Flavonoids. Oxid. Med. Cell. Longev..

[38] Žuvela P., David J., Yang X., Huang D., Wong M.W. (2019). Non-Linear Quantitative Structure-Activity Relationships Modelling, Mechanistic Study and in-Silico Design of Flavonoids as Potent Antioxidants. Int. J. Mol. Sci..

[39] Pietta P.G. (2000). Flavonoids as Antioxidants. J. Nat. Prod..

[40] Lee J.H., Kim Y., Hoang M.H., Jun H.J., Lee S.J. (2014). Rapid Quantification of Cellular Flavonoid Levels Using Quercetin and a Fluorescent Diphenylboric Acid 2-Amino Ethyl Ester Probe. Food Sci. Biotechnol..

[41] Hostetler G., Riedl K., Cardenas H., Diosa-Toro M., Arango D., Schwartz S., Doseff A.I. (2012). Flavone Deglycosylation Increases Their Anti-Inflammatory Activity and Absorption. Mol. Nutr. Food Res..

[42] Grootaert C., Gonzales G.B., Vissenaekens H., Van De Wiele T., Raes K., Smagghe G., Van Camp J. (2016). Flow Cytometric Method for the Detection of Flavonoids in Cell Lines. J. Biomol. Screen..

[43] De R., Mahata M.K., Kim K. (2022). Structure-Based Varieties of Polymeric Nanocarriers and Influences of Their Physicochemical Properties on Drug Delivery Profiles. Adv. Sci..

[44] Anouar E.H., Gierschner J., Duroux J.L., Trouillas P. (2012). UV/Visible Spectra of Natural Polyphenols: A Time-Dependent Density Functional Theory Study. Food Chem..

[45] Tristantini D., Amalia R. (2019). Quercetin Concentration and Total Flavonoid Content of Anti-Atherosclerotic Herbs Using Aluminum Chloride Colorimetric Assay. AIP Conf. Proc..

[46] Szkop M., Kliszcz B., Kasprzak A.A. (2018). A Simple and Reproducible Protocol of Glass Surface Silanization for TIRF Microscopy Imaging. Anal. Biochem..

[47] De R., Song Y.H., Mahata M.K., Lee K.T. (2022). pH-Responsive Polyelectrolyte Complexation on Upconversion Nanoparticles: A Multifunctional Nanocarrier for Protection, Delivery, and 3D-Imaging of Therapeutic Protein. J. Mater. Chem. B.

[48] Salucci M., Bugianesi R., Maiani G., Stivala L.A., Vannini V. (2002). Flavonoids Uptake and Their Effect on Cell Cycle of Human Colon Adenocarcinoma Cells (CaCO_2_). Br. J. Cancer.

[49] Oh J.M., Kim E., Chun S. (2019). Ginsenoside Compound K Induces Ros-Mediated Apoptosis and Autophagic Inhibition in Human Neuroblastoma Cells in Vitro and in Vivo. Int. J. Mol. Sci..

[50] Zsila F., Bikádi Z., Simonyi M. (2003). Probing the Binding of the Flavonoid, Quercetin to Human Serum Albumin by Circular Dichroism, Electronic Absorption Spectroscopy and Molecular Modelling Methods. Biochem. Pharmacol..

[51] Singh R., Wu B., Tang L., Liu Z., Hu M. (2010). Identification of the Position of Mono-O-Glucuronide of Flavones and Flavonols by Analyzing Shift in Online UV Spectrum (Λmax) Generated from an Online Diode Array Detector. J. Agric. Food Chem..

[52] Marby T.J., Markham K.R., Thomas M.B. (1970). The Systematic Identification of Flavonoids.

[53] Matteini P., Agati G., Pinelli P., Goti A. (2011). Modes of Complexation of Rutin with the Flavonoid Reagent Diphenylborinic Acid 2-Aminoethyl Ester. Mon. Fur Chem..

[54] Katyal M., Ryan D.E. (1969). Fluorescence Test for Flavonols. Anal. Lett..

[55] Biler M., Biedermann D., Valentová K., Křen V., Kubala M. (2017). Quercetin and Its Analogues: Optical and Acido-Basic Properties. Phys. Chem. Chem. Phys..

[56] Hollman P.C.H., Van Trijp J.M.P., Buysman M.N.C.P. (1996). Fluorescence Detection of Flavonols in HPLC by Postcolumn Chelation with Aluminum. Anal. Chem..

[57] Höfener S., Kooijman P.C., Groen J., Ariese F., Visscher L. (2013). Fluorescence Behavior of (Selected) Flavonols: A Combined Experimental and Computational Study. Phys. Chem. Chem. Phys..

[58] Seyoum A., Asres K., El-Fiky F.K. (2006). Structure-Radical Scavenging Activity Relationships of Flavonoids. Phytochemistry.

[59] Ramírez-Moreno I.G., Ibarra-Sánchez A., Castillo-Arellano J.I., Blank U., González-Espinosa C. (2020). Mast Cells Localize in Hypoxic Zones of Tumors and Secrete CCL-2 under Hypoxia through Activation of L-Type Calcium Channels. J. Immunol..

