# Supplementary material for: Influence of Molecular Structures on Fluorescence of Flavonoids and Their Detection in Mammalian Cells
Source: Biomedicines. 2022 May 28;10(6):1265. doi: 10.3390/biomedicines10061265 (PMC9220233; doi:10.3390/biomedicines10061265)
Supplement: Supplementary file 1 [file biomedicines-10-01265-s001.zip › biomedicines-1688480-supplementary.pdf]

Supplementary Information

Influence of Molecular Structures on Fluorescence of Flavonoids and Their Detection in Mammalian Cells

Ranjit De, Kyung Won Jo, and Kyong-Tai Kim*

Laboratory of Molecular Neurophysiology, Department of Life Sciences, Pohang University of Science and Technology (POSTECH), Pohang 37673, South Korea

*Corresponding Author. ktk@postech.ac.kr (KTK)

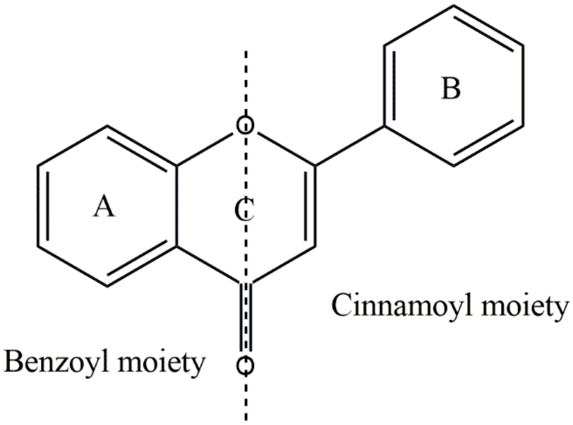

Figure S1. A schematic presentation of the basic flavonol structure comprised of the benzoyl and cinnamoyl moieties.

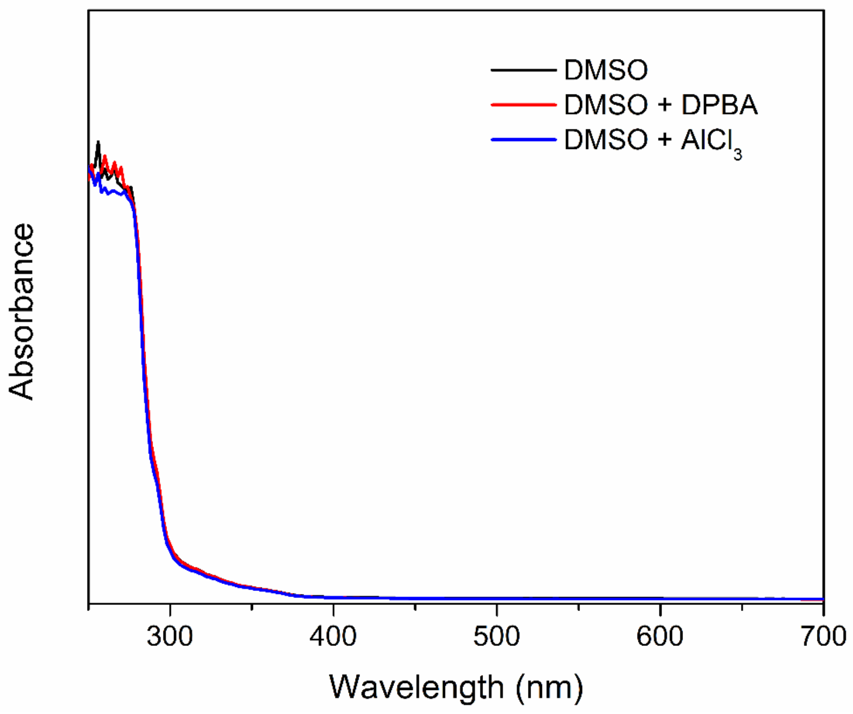

Figure S2. UV-visible spectra of DPBA and $AlCl_3$ in DMSO showing no peaks in the absence of flavonoids.

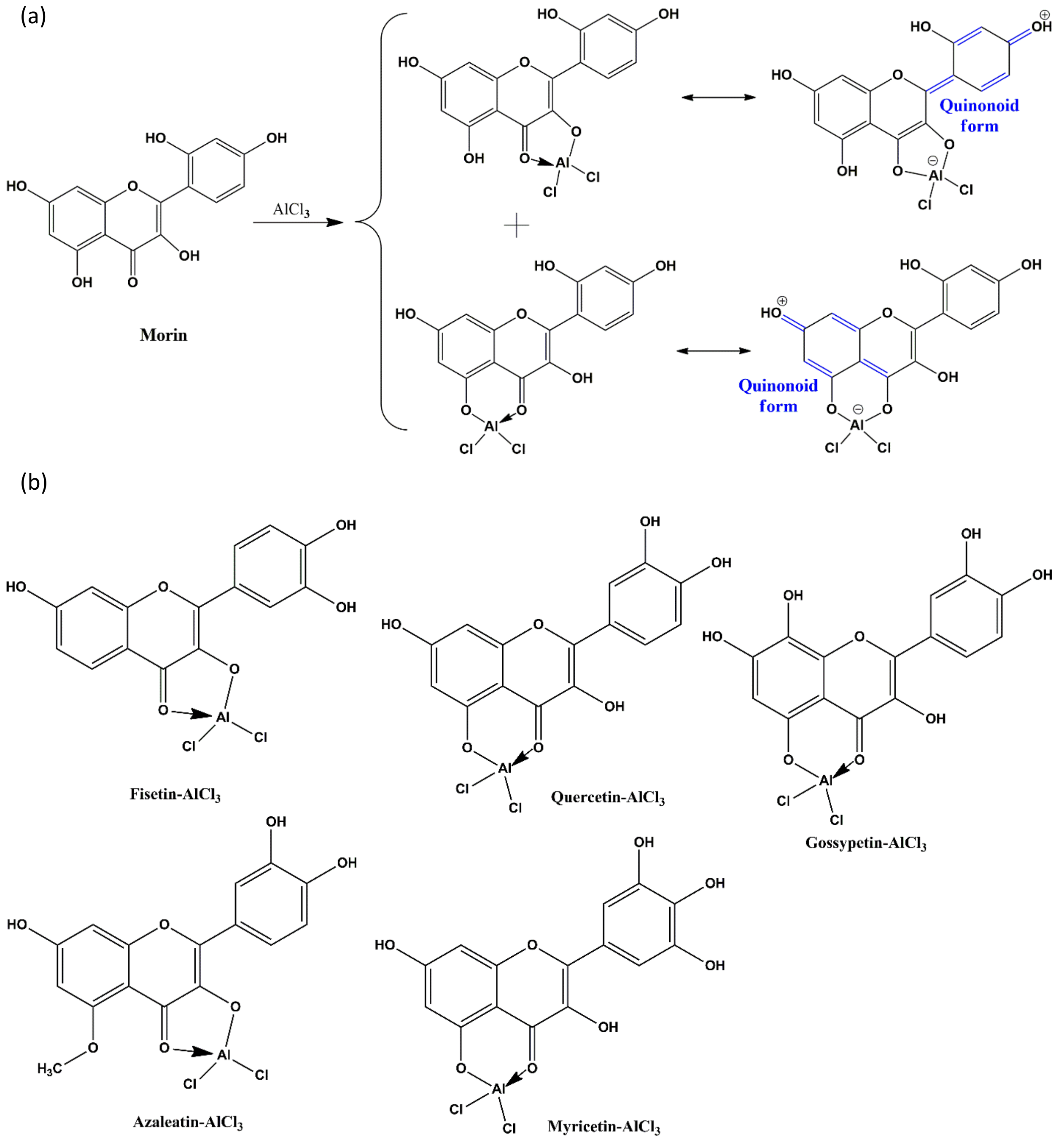

Figure S3. (a) A schematic presentation of the complexation of $AlCl_3$ with morin and the plausible quinonoid forms. (b) The plausible molecular structures of complexes formed on treatment of fisetin, quercetin, gossypetin, azaleatin, and myricetin with $AlCl_3$.

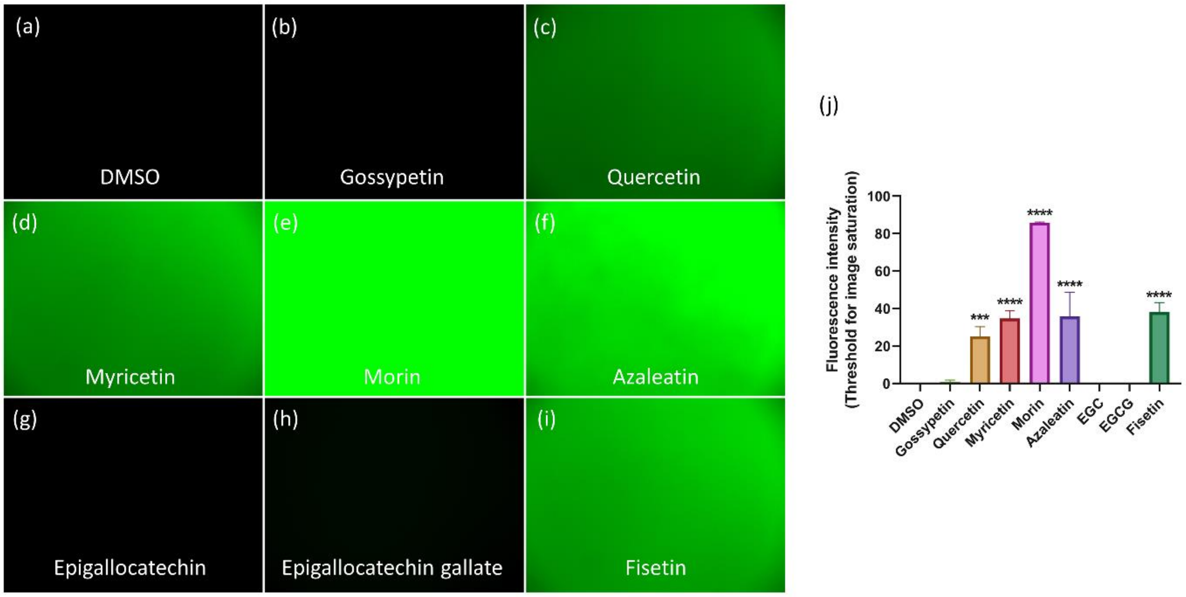

Figure S4. *In vitro* Investigation of fluorescence intensity of the flavonoids after chelation with $AlCl_3$ (a-i) and a comparison of their relative intensity (j). The error bars represent the mean ± SEM. ****P < 0.0001, ***P < 0.001, one-way ANOVA followed by Tukey's multiple comparisons test (j).

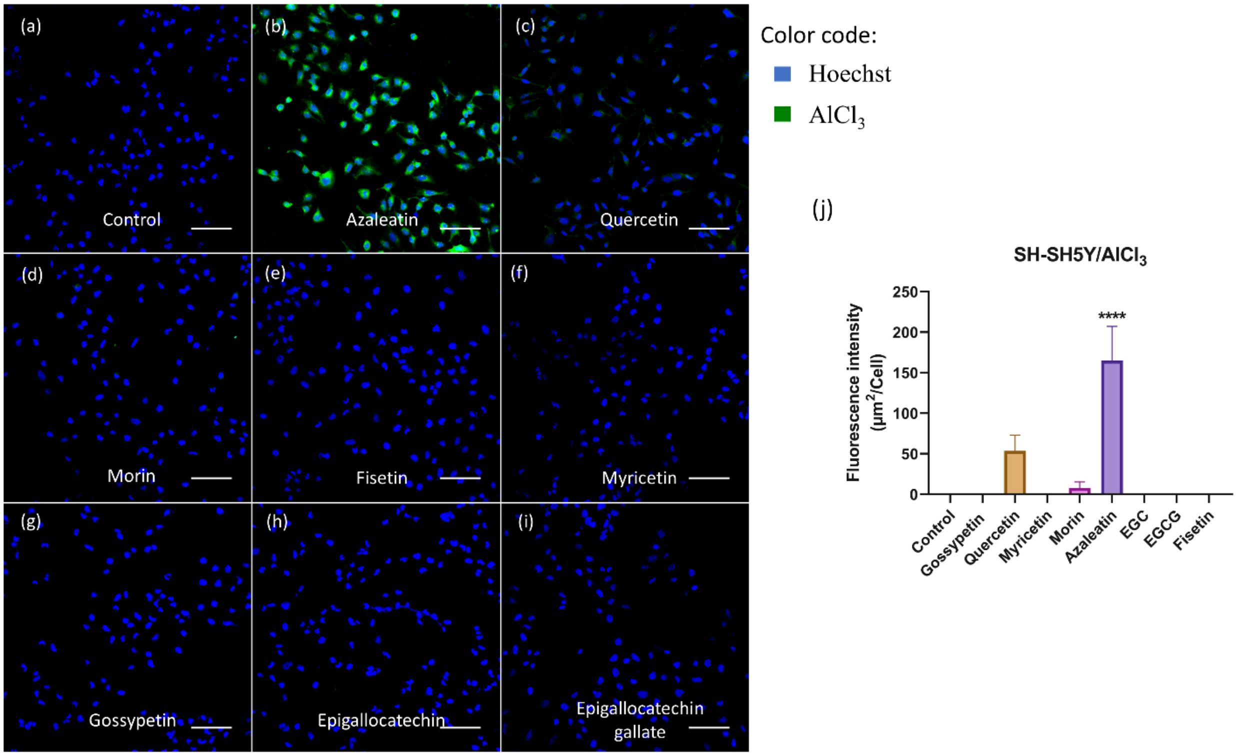

Figure S5. Fluorescence microscopic images of human neuroblastoma (SH-SY5Y) cells after flavonoid uptake followed by $AlCl_3$ treatment. Cells are treated with (a) DMSO (flavonoid absent, control), (b) azaleatin, (c) quercetin, (d) fisetin, (e) morin, (f) myricetin, (g) gossypetin, (h) epigallocatechin, and (i) epigallocatechin gallate. Flavonoid treatment (100 μM) is carried out for 1 h, while the enhancer $AlCl_3$ solution (0.01 M in water) is employed for 10 min. The scale bars represent 100 μm. Nucleus was stained using Hoechst. Among these, the azaleatin, quercetin, fisetin and morin treated cells showed fluorescence while it was not detectable in gossypetin, epigallocatechin, and epigallocatechin gallate treated cell. Panel (j) represents a comparison of fluorescence intensity exhibited by the flavonoid treated cells. The error bars represent the mean ± SEM. ****P < 0.0001, one-way ANOVA followed by Tukey’s multiple comparisons test (j).

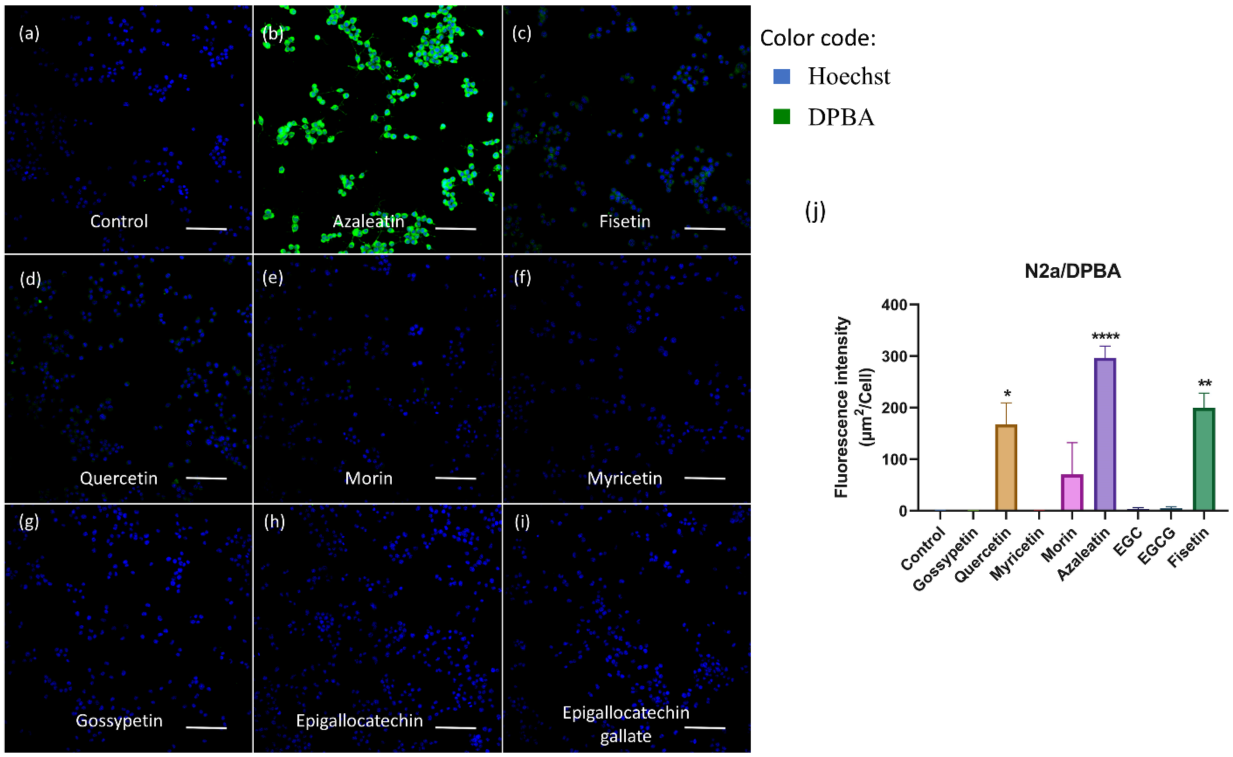

Figure S6. Fluorescence microscopic images of mouse neuroblastoma (N2a) cells after flavonoid uptake followed by DPBA treatment. Cells are treated with (a) DMSO (flavonoid absent, control), (b) azaleatin, (c) quercetin, (d) fisetin, (e) morin, (f) myricetin, (g) gossypetin, (h) epigallocatechin, and (i) epigallocatechin gallate. Flavonoid treatment (100 μM) is carried out for 1 h, while the enhancer DPBA solution (0.5% wt./v, in ethanol) is employed for 10 min. The scale bars represent 100 μm. Nucleus was stained using Hoechst. Among these, the azaleatin, quercetin, fisetin and morin treated cells showed fluorescence while it was not detectable in gossypetin, epigallocatechin, and epigallocatechin gallate treated cell. Panel (j) represents a comparison of fluorescence intensity exhibited by the flavonoid treated cells. The error bars represent the mean ± SEM. ****$P < 0.0001$, **$P < 0.01$, *$P < 0.05$, one-way ANOVA followed by Tukey's multiple comparisons test (j).

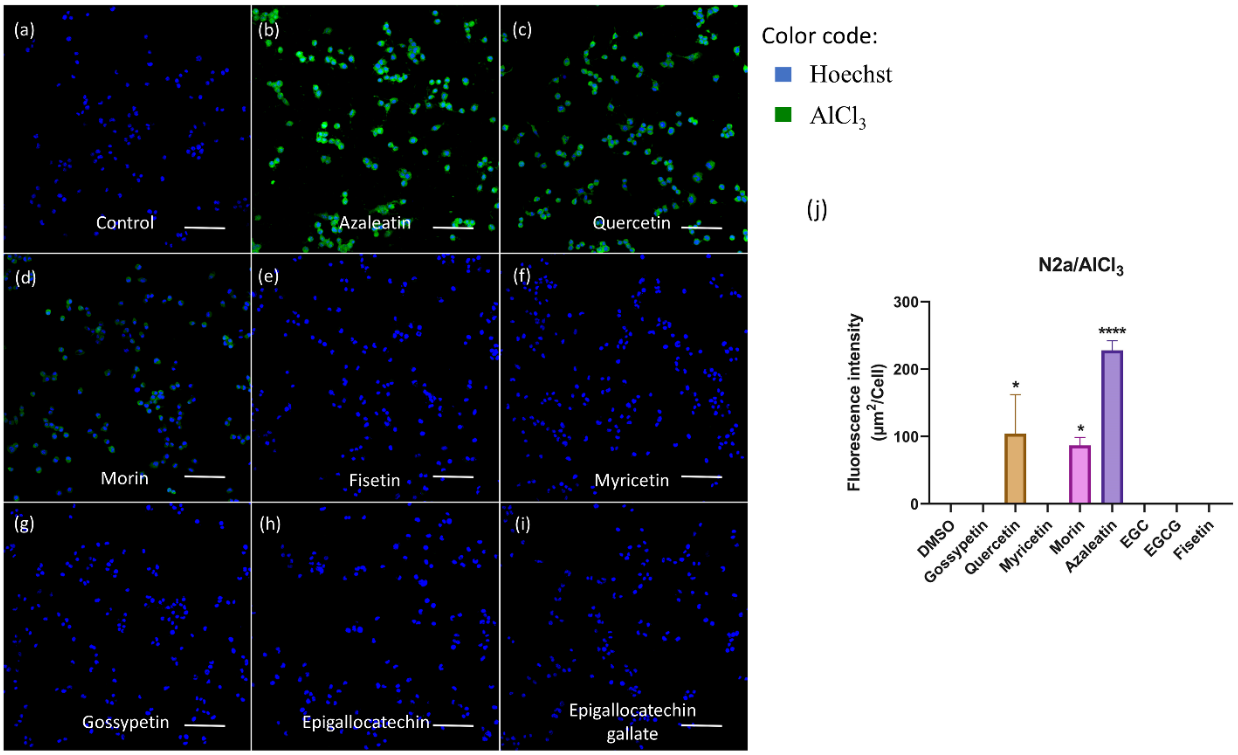

Figure S7. Fluorescence microscopic images of human neuroblastoma (N2a) cells after flavonoid uptake followed by $AlCl_3$ treatment. Cells are treated with (a) DMSO (flavonoid absent, control), (b) azaleatin, (c) quercetin, (d) fisetin, (e) morin, (f) myricetin, (g) gossypetin, (h) epigallocatechin, and (i) epigallocatechin gallate. Flavonoid treatment (100 μM) is carried out for 1 h, while the enhancer $AlCl_3$ solution (0.01 M in water) is employed for 10 min. The scale bars represent 100 μm. Nucleus was stained using Hoechst. Among these, the azaleatin, quercetin, fisetin and morin treated cells showed fluorescence while it was not detectable in gossypetin, epigallocatechin, and epigallocatechin gallate treated cell. Panel (j) represents a comparison of fluorescence intensity exhibited by the flavonoid treated cells. The error bars represent the mean ± SEM. ****$P < 0.0001$, *$P < 0.05$, one-way ANOVA followed by Tukey's multiple comparisons test (j).

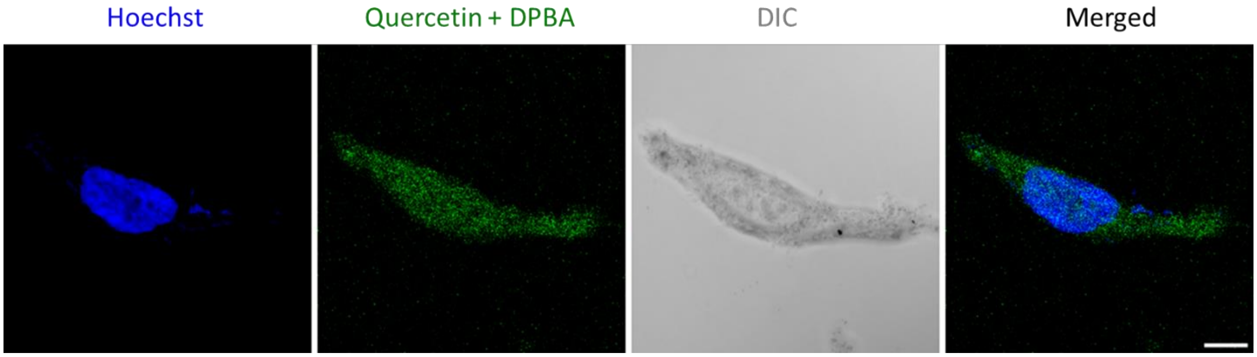

Figure S9. A magnified image of quercetin treated SH-SY5Y single cell stained with DPBA showing the distribution of quercetin inside the cell. The scale bar represents 10 μm.
